# Targeting the Wnt/β-catenin pathway in endometriosis: a potentially effective approach for treatment and prevention

**DOI:** 10.1186/s40591-014-0036-9

**Published:** 2014-11-19

**Authors:** Sachiko Matsuzaki, Revaz Botchorishvili, Jean Luc Pouly, Michel Canis

**Affiliations:** CHU Clermont-Ferrand, CHU Estaing, Chirurgie Gynécologique, 1, Place Lucie et Raymond Aubrac, 63003 Clermont-Ferrand, France; Clermont Université, Université d’Auvergne, ISIT UMR6284, Clermont-Ferrand, France; CNRS, ISIT UMR6284, Clermont-Ferrand, France

**Keywords:** Endometriosis, Endometrium, Wnt/β-catenin pathway

## Abstract

Endometriosis is a chronic, estrogen-dependent disease associated with infertility and pelvic pain. Endometriosis is defined by the presence of extra-uterine endometrial tissue. It affects approximately 10% of reproductive-aged women. However, the underlying etiology, pathogenesis and pathophysiology remain to be fully elucidated. Knowledge of these factors is indispensable for the development of targeted therapies for prevention and treatment of endometriosis. Several studies, including those from our laboratory, have suggested that aberrant activation of the Wnt/β-catenin pathway may be involved in the pathophysiology of endometriosis. This is a review of the literature focused on the aberrant activation of the Wnt/β-catenin pathway in patients with endometriosis, and on how targeting the Wnt/targeting pathway may be a potentially effective approach for treating and/or preventing endometriosis.

## Background

Endometriosis is a chronic, estrogen-dependent disease associated with infertility and pelvic pain. Endometriosis is defined by the presence of extra-uterine endometrial tissue. It affects approximately 10% of reproductive-aged women [1]. However, the underlying etiology, pathogenesis and pathophysiology remain to be fully elucidated. Knowledge of these factors is indispensable for the development of targeted therapies for prevention and treatment of endometriosis.

**Figure 1:**
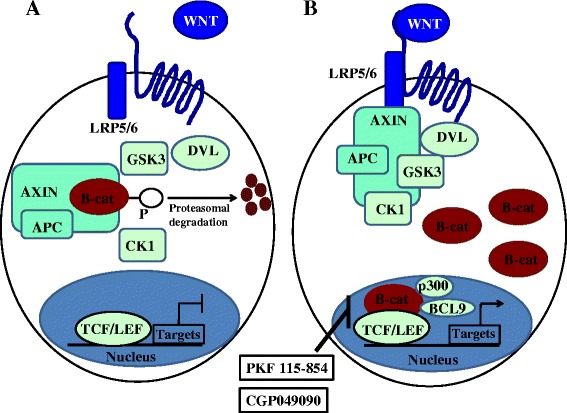
**The Wnt/β-catenin signaling pathway. A)** In the absence of Wnt ligands such as Wnt1, Wnt3a, and Wnt8 (“off” state), β-catenin is degraded by the APC/Axin/GSK-3ß complex. **B)** Binding of Wnt ligands to the Frizzled transmembrane receptors and their LRP co-receptors (“on” state) leads to the inactivation of GSK-3ß and accumulation of β-catenin in the cytoplasm. Then, the elevated cytosolic β-catenin can translocate to the nucleus, where it interacts with the Tcf/LEF transcription factors, leading to transcriptional activation of Wnt-responsive genes. Many Wnt-responsive genes have crucial roles in cell proliferation, migration, and invasion. Two fungal derivatives (PKF 115–854 and CGP049090), small-molecule antagonists of the Tcf/β-catenin complex, disrupt the critical protein-protein interaction between β-catenin and Tcf as indicated in this figure. Effects of PKF 115–854 and/or CGP049090 on endometriosis are summarized in Table 1. APC: adenomatous polyposis coli, GSK-3ß: glycogen synthase kinase 3β, LRP: lipoprotein receptor-related protein, TCF/LEF: T-cell factor/lymphocyte enhancer factor, CK1: casein kinase, DVL: disheveled.

Several studies, including those from our laboratory, have suggested that aberrant activation of the Wnt/β-catenin pathway may be involved in the pathophysiology of endometriosis [2–9]. The Wnt/β-catenin pathway has crucial roles in embryonic development, tissue self-renewal, and various diseases [10–13]. In the absence of Wnt ligands (“off” state), β-catenin is degraded by the APC/Axin/GSK-3ß complex [10–13] (Figure 1A). Binding of Wnt ligands to the Frizzled transmembrane receptors and their LRP co-receptors (“on” state) leads to the inactivation of GSK-3ß and accumulation of β-catenin in the cytoplasm. Then, the elevated cytosolic β-catenin can translocate to the nucleus, where it interacts with the Tcf/LEF transcription factors, leading to transcriptional activation of Wnt-responsive genes (Figure 1B). Many Wnt-responsive genes have crucial roles in cell proliferation, migration, and invasion [10–13]. These processes are also common in endometriosis [1].

This is a review of the literature focused on the aberrant activation of the Wnt/β-catenin pathway in patients with endometriosis, and on how targeting the Wnt/targeting pathway may be a potentially effective approach for treating and/or preventing endometriosis.

## Review

### Aberrant activation of the Wnt/β-catenin pathway in endometrium of patients with endometriosis

#### Menstrual endometrium of patients with endometriosis

Pathogenesis of endometriosis remains unclear. However, the implantation theory is the most widely accepted [1]. Endometriosis originates from retrograde menstruation of endometrial tissue that passes through patent Fallopian tubes into the peritoneal cavity. As retrograde menstruation is a common physiological event, it remains unknown why endometriosis only occurs in about 10% of women during their reproductive years. One possible explanation is that the eutopic endometrial cells of women with endometriosis may be functionally and biochemically different from those of women without endometriosis [1].

Our previous study showed significantly higher total and active forms of MMP-9 in the menstrual epithelial and stromal cells of patients with endometriosis compared to those of patients without endometriosis [4]. MMP-9 is one of the Tcf/β-catenin target genes (www.stanford.edu/group/nusselab/cgi-bin/wnt/target_genes). Treatment with PKF 115–584, a small-molecule antagonist of the Tcf/β-catenin complex, decreased the amount of total MMP-9 approximately 75% in epithelial cells and 85% in stromal cells in patients with endometriosis [4]. Furthermore, treatment with PKF 115–584 decreased the amount of active MMP-9 to undetectable levels in both epithelial and stromal cells of patients with endometriosis [4]. MMP-9 activity is known to be involved in cell invasion [14–17]. In addition, recent studies clearly demonstrated that a latent form of MMP-9 may play an important role in cell migration [18,19]. Our previous study demonstrated that the inhibitory effects of cell migration and invasion of menstrual endometrial epithelial and stromal cells of endometriosis patients by treatment with PKF 115–584 were much higher than those of patients without endometriosis [4]. These findings are consistent with those of a previous study that demonstrated that MMP-9 secretion, as assessed by zymography and enzyme-linked immunosorbent assay (ELISA), was increased in women with endometriosis compared to healthy women [20]. According to the implantation theory, two processes appear to be critical for the establishment of endometriosis: migration and invasion [1,21]. These findings suggested that aberrant activation of the Wnt/β-catenin pathway may result in increased migration and invasion of menstrual endometrial cells of patients with endometriosis.

Furthermore, a recent study showed that human endometrial basal glandular epithelial cells expressed nuclear SOX9, a Wnt target gene, and contained a rare subpopulation of cells with nuclear β-catenin [22]. These findings suggested that the Wnt pathway was activated in the basal endometrium. They also showed that the embryonic stem cell-surface marker, SSEA-1, marked the human endometrial basal glandular epithelial cells. Interestingly, cells in ectopic endometriosis lesions also expressed SSEA-1 and nuclear SOX9, and SOX9 and SSEA-1 expression patterns were similar to those in the matched eutopic basalis epithelia [22]. These investigators speculated that the ectopic lesions might be basalis in origin following retrograde menstruation [22]. A growing body of evidence suggests that endometriosis may arise from stem cells [23–27]. The Wnt signaling pathway plays an essential role in stem cell regulation [13]. Although further studies are required to examine the role of the Wnt/β-catenin signaling in SOX9– and SSEA-1–expressing endometrial glandular epithelial cells, these findings suggested that manipulation of Wnt signaling for stem cell regulation might be a novel therapeutic strategy for prevention and treatment of endometriosis.

#### Mid-secretory endometrium of infertile patients with endometriosis

Endometriosis affects approximately 25%-50% of all women with infertility [1]. However, the underlying molecular mechanisms of endometriosis-associated infertility remain to be elucidated. One potential cause may be endometrial molecular defects during the implantation window [1].

Previous findings, including those from our laboratory, suggested that the Wnt/beta-catenin signaling pathway might be aberrantly activated in infertile patients during the mid-secretory phase [2,6,7]. Our previous study demonstrated punctate membranous expression of dephosphorylated beta-catenin in endometrial epithelial cells of infertile patients during the mid-secretory phase [2]. Expression of the N-terminally dephosphorylated form of beta-catenin has been shown to be well correlated with Wnt activity [28]. Our previous study showed significantly higher basal cell proliferation of endometrial epithelial and stromal cells of patients with endometriosis compared to patients without endometriosis in the mid-secretory phase [4]. In addition, mRNA expression of Cyclin D1, a Tcf/β-catenin target gene, was significantly higher in endometrial epithelial cells of patients with endometriosis compared to patients without endometriosis in the mid-secretory phase [4]. Expression levels of Cyclin D1 tended to be higher in the secretory phase stromal cells of patients with endometriosis compared to patients without endometriosis, which is in agreement with with the results of a previous study [29]. Cell proliferation and Cyclin D1 mRNA expression in epithelial and stromal cells of patients with endometriosis were effectively decreased by treatment with PKF 115–584, a small-molecule antagonist of the Tcf/β-catenin complex.

A study showed that increased E2 levels activate Wnt/β-catenin signaling to promote endometrial cell proliferation during the proliferative phase of menstrual cycle, whereas during the secretory phase, progesterone levels inhibit Wnt/β-catenin signaling, resulting in counterbalancing E2-induced proliferation and enhancing differentiation [30]. In addition, a mouse study demonstrated that stabilization of beta-catenin in the uterus resulted in endometrial glandular hyperplasia and lack of a decidual response [31]. In these mice, estrogen receptor (ER)-alpha expression was increased in the epithelium [31]. A growing body of evidence suggests that endometrium of patients with endometriosis has an altered response to progesterone and persistence of the proliferative phenotype [7,29,32]. Studies have demonstrated impaired decidualization in endometrial stromal cells and elevated endometrial ER-alpha expression during the mid-secretory phase in patients with endometriosis [33]. Furthermore, regulation of cyclin D1, one of the target genes of the Wnt pathway via activation of β-catenin, is impaired in endometrial stromal cells of patients with endometriosis [7]. These findings suggest that progesterone resistance might fail to inhibit activation of Wnt/β-catenin signaling, resulting in the persistence of the proliferative phenotype and impaired decidualization in the endometrium of infertile patients with endometriosis during the window of implantation.

### Aberrant activation of the Wnt/β-catenin pathway in endometriosis

#### Cell proliferation, migration and invasion of endometriotic cells

The Wnt/beta-catenin pathway is involved in cell proliferation, migration, and invasion [34], which are also involved in the pathophysiology of endometriosis [1]. Our previous study showed that PKF 115–584, a small-molecule antagonist of the Tcf/β-catenin complex, could significantly decrease cell proliferation, migration and invasion of endometrial and endometriotic epithelial and stromal cells [4]. However, our previous findings suggested that the Wnt/β-catenin signaling pathway might not be essential for cell proliferation of endometriotic cells [4]. The inhibitory effect of treatment with PKF 115–584 on cell proliferation in ovarian endometriotic tissue was significantly lower than that of matched eutopic endometrium of the same patients [4]. The cell proliferation inhibitory effect of deep infiltrating endometriotic tissue and superficial peritoneal endometriotic tissue tended to be lower than that of matched eutopic endometrium of the same patients [4]. Furthermore, we showed that in either non-treated or treated cells with PKF 115–584, no significant difference in the number of migrated epithelial and stromal cells was observed between endometriotic tissue and matched eutopic endometrium of the same patients [4]. In addition, we observed that no significant difference in percent inhibition of cell migration by treatment with PKF 115–584 in either epithelial or stromal cells was noted between endometriotic tissue and eutopic endometrium of the same patients [4]. Activation of the Wnt/β-catenin pathway may not be as involved in the cell proliferation and migration of diseased cells—endometriotic cells—than their normal cell counterparts—endometrial cells.

In contrast, our previous study demonstrated that endometriotic epithelial cells and stromal cells were more invasive than those of matched eutopic endometrium of the same patients [4]. These findings are in agreement with the results of previous in vitro experiments studies that showed that endometriotic cells have invasive and metastatic phenotypes similar to metastatic carcinoma cells [35,36]. The numbers of invasive endometriotic epithelial and stromal cells were effectively decreased by treatment with PKF 115–584 [4]. Levels of active MMP-2 in endometriotic epithelial cells and total and active MMP-9 in endometriotic stromal cells were significantly decreased compared to those of matched eutopic endometrium following treatment with PKF 115–584 [4]. Both MMP-9 and MMP-2 are Tcf/β-catenin target genes [4]. These findings suggested that inhibition of active MMP2 and MMP9 by treatment with PKF 115–584 decreased the numbers of invasive endometriotic epithelial cells and stromal cells. Aberrant activation of the Wnt/β-catenin signaling pathway may be involved in the invasive phenotype of endometriotic cells.

Furthermore, a recent study demonstrated that the activated TNF­α–MMP-9-SRC-1 axis protects the ectopic endometrium from proinflammatory cytokine­mediated apoptosis [37]. Endometriotic cells exhibit abnormal apoptotic regulation [38]. Aberrant activation of the Wnt/β-catenin signaling pathway might also be involved in resistance of endometriotic stromal cells to apoptosis through the TNF­α–MMP-9 axis.

Recent studies demonstrated the presence of ectopic endometrium in the rectovaginal septum, in the Douglas pouch, in the rectum of human female fetuses [39,40]. They hypothesized that ectopic endometrium might be misplaced outside the uterine cavity during the organogenesis [39,40]. The Wnt/beta-catenin signaling pathway is essential for organogenesis [41]. Endometriosis in some patients may arise from müllerian duct remnants. The Wnt/beta-catenin signaling pathway may possibly be activated by hormonal inputs in the ectopic endometrium after puberty starts [30,42]. However, it is unlikely that all endometriosis could arise from müllerian duct remnants, because the distribution of pelvic endometriosis differs from that of embryonic duct remnants.

#### Fibrosis in endometriosis

Endometriosis is histologically characterized by dense fibrous tissue mainly composed of collagen type I [1,43]. Excess fibrosis may cause severe clinical symptoms, such as pelvic pain, severe dysmenorrhea, and deep dyspareunia in patients with endometriosis [44,45], Endometriosis is an estrogen-dependent disease. However, hormonal suppressive therapy is not usually effective for deep infiltrating endometriosis [45]. Complete surgical removal of the deep endometriotic lesions results in the best long-term results and symptomatic relief [45]. However, in addition to the dense fibrosis, deep infiltrating endometriosis frequently invades vital pelvic organs [45]. Surgical treatment of deep infiltrating endometriosis should be performed by laparoscopic surgeons who are highly skilled and competent in performing bowel, bladder, and ureteral surgery [45].

Despite of its clinical importance, only a few studies have been conducted to evaluate new therapies for fibrosis in endometriosis. The cellular and molecular mechanisms underlying fibrosis in endometriosis remain to be fully elucidated.

Recent studies have demonstrated the involvement of activated Wnt/β-catenin signaling in fibrosis in several organs [46–50]. Our previous study showed that Wnt3a treatment in the endometrial stromal cells of patients without endometriosis significantly increased cell proliferation and migration, cell-mediated contraction of collagen gels, and expression of fibrotic marker genes (alpha-smooth muscle actin, type I collagen, connective tissue growth factor, and fibronectin) [5]. We showed a significantly lower cell-mediated collagen gel contraction in stromal cells from patients without endometriosis compared to patients with endometriosis [5]. Cell-mediated contraction of collagen gel in stromal cells of patients without endometriosis was increased by treatment with Wnt3a to a level comparable with that of patients with endometriosis [5]. Treatment with Wnt3a induced clearly visible αSMA-positive stress fibers, the hallmark of activated myofibroblasts, in endometrial stromal cells of patients without endometriosis. These findings suggested the involvement of the aberrant activation of the Wnt/β-catenin pathway in the molecular and cellular mechanisms underlying fibrogenesis of endometriosis [5]. Further studies are required whether overexpression of Wnt3a is one of the underlying mechanisms for the development of fibrosis in endometriosis.

Furthermore our previous study demonstrated that mRNA expression of fibrotic marker genes was significantly decreased by treatment with PKF 115–584 and CGP049090, small-molecule antagonists of the Tcf/β-catenin complex [5]. Treatment with PKF 115–584 and CGP049090 significantly decreased stromal cell-mediated contraction of collagen gels in endometrium and endometriosis [5]. Our previous study also showed that cell proliferation and migration of endometrial and endometriotic stromal cells were significantly decreased by treatment with PKF 115–584 [4]. Fibroblast migration, proliferation, and collagen contraction are main hallmarks of fibrogenesis [51]. Small-molecule antagonists of the Tcf/β-catenin complex may inhibit fibrogenesis in endometriosis [4,5]. Furthermore, we showed that treatment with CGP049090 prevented the progression of fibrosis in a xenograft model of endometriosis in nude mice [5]. More importantly, we observed that treatment with CGP049090 revresed established fibrosis in our mouse model of endometriosis [5]. A study demonstrated that treatment with ICG-001, a selective inhibitor of Wnt/β-catenin-CBP-dependent transcription, prevented and reversed fibrosis in a mouse model of bleomycin-induced pulmonary fibrosis [49]. These findings and our findings support that aberrant activation of the Wnt/β-catenin signaling pathway play an important role in the pathogenesis of fibrosis.

### Chronic pelvic pain in endometriosis: potential involvement of aberrant activation of the Wnt/β-catenin pathway

Pain is a major clinical problem in patients with endometriosis [1,52–55]. However, the underlying mechanisms are not yet very well understood. The group of Berkley demonstrated in endometriosis is associated with central sensitization that underlies pain hypersensitivity [54]. In addition, the same group recently proposed that painful endometriosis can be classified as a mixed inflammatory/neuropathic pain condition [55]. Thus, therapeutic strategies for neuropathic pain might be applied to endometriosis [53,55]. Central sensitization-associated synaptic plasticity in the spinal cord dorsal horn (SCDH) critically contributes to the development of chronic pain [52,53]. A recent study showed that both Wnt3a and β-catenin are up-regulated in the SCDH of various mouse pain models: the capsaicin pain model, the HIV-gp120 pain model, and the neuropathic pain model [56]. These results suggested that Wnt signaling pathways are regulated by nociceptive input [56]. The activation of Wnt signaling may contribute to the spinal cord central sensitization [56]. In addition, a recent animal experiment showed that nerve injury caused rapid-onset and long-lasting expression of Wnts and activation of Wnt/Frizzled/β-catenin signaling in primary sensory neurons, SCDH neurons, and astrocytes [57]. Furthermore, Wnt signaling activation stimulated production of the proinflammatory cytokines IL-18 and TNF-α, which play an important role in the generation of neuropathic pain, through the β-catenin pathway in the spinal cord [57]. Blocking Wnt/β-catenin signaling may provide a strategy for treating neuropathic pain through IL-18 and TNF-α inhibition [56]. Although to date no studies have investigated the activation of Wnt signaling in the SCDH in animal models of endometriosis pain, previous animal experiments suggested the potential involvement of Wnt signaling activation in the molecular mechanisms that underlie chronic pelvic pain in endometriosis.

## Conclusions

Studies, including those from our laboratory, suggested that the aberrant activation of the Wnt/β-catenin pathway may be involved in the pathophysiology of endometriosis. The aberrant activation of the Wnt/β-catenin pathway in menstrual endometrium may facilitate development of endometriosis through increased cell migration and invasion.

In addition, the aberrant activation of the Wnt/β-catenin pathway may facilitate growth of endometriosis through an increased invasive phenotype and resistance to apoptosis of endometriotic cells. Furthermore, the aberrant activation of the Wnt/β-catenin pathway in mid-secretory endometrium may result in the persistence of the proliferative phenotype and impaired decidualization in infertile patients with endometriosis. The findings from our laboratory also showed the involvement of the Wnt/β-catenin signaling pathway in the cellular and molecular mechanisms underlying fibrosis in endometriosis. A small-molecule antagonist of the Tcf/β-catenin complex prevented the progression of fibrosis and, more importantly, reversed established fibrosis in a xenograft model of endometriosis in immunodeficient nude mice. These findings suggested that targeting the Wnt signaling pathway may be a potentially effective approach for treating and/or preventing endometriosis (Table 1).

**Table 1:** **Summary of effects of small-molecule antagonists of the Tcf/β-catenin complex on endometriosis**

**Model**	**Cell type or species**	**Inhibitor**	**Parameters assessed**	**Functional effects**	**Reference**
In vitro assay	C-EE, C-ES	PKF 115-584	Cell proliferation	C-EE, C-ES, E-EE, E-ES, EnE and EnS: significantly inhibited vs. non-treated cells	[4]
	E-EE, E-ES	CGP049090			
	EnE, EnS				
	C-EE, C-ES	PKF 115-584	Cell migration	C-EE, C-ES, E-EE, E-ES, EnE and EnS: significantly inhibited vs. non-treated cells	[4]
	E-EE, E-ES				
	EnE, EnS				
	C-EE, C-ES	PKF 115-584	Cell invasion	C-EE, C-ES, E-EE, E-ES, EnE and EnS: significantly inhibited vs. non treated cells	[4]
	E-EE, E-ES				
	EnE, EnS				
	C-EE, C-ES	PKF 115-584	Tcf/β-catenin target genes (Cyclin D1, Survivin, c-Myc, MMP2, MMP9)	C-EE, C-ES, E-EE, E-ES, EnE and EnS: cyclin D1, Survivin, MMP2 and MMP9 mRNA: significantly decreased vs. non-treated cells	[4]
	E-EE, E-ES				
	EnE, EnS				
				Total form of MMP-9 in E-EE or E-ES: significantly decreased vs. C-EE or C-ES	
				Active forms of MMP-2 in EnE: significantly decreased vs. E-EE	
				Total form of MMP-9 in EnS: significantly decreased vs. E-ES	
	E-ES, EnS	PKF 115-584	Fibrotic markers (αSMA, Col-I, CTGF, and FN)	E-ES, EnS: αSMA, Col-I, CTGF, and FN mRNA (with or without TGF ß1 stimulation): significantly decreased vs. non-treated cells	[4]
		CGP049090		EnS: percentage of αSMA-positive cells: significantly decrased vs. non-treated cells	
	E-ES, EnS	PKF 115-584	Collagen gel contraction	E-ES, EnS: significantly decreased vs. non-treated cells	[5]
		CGP049090			
In vivo assay	Mouse (female Swiss nude mice)	CGP049090	Severity of fibrosis in endometriotic implants assessed by Sirius Red or Masson Trichrome stains	Staining scores for Sirius Red or Masson Trichrome stains: significantly lower vs. non-treated mice	[5]

C-EE: endometrial epithelial cells of patients without endometriosis, C-ES: endometrial stromal cells of patients without endometriosis.

E-EE: Endometrial epithelial cell of patients with endometriosis, E-ES: endometrial stromal cell of patients with endometriosis.

EnE: Endometriotic epithelial cells, EnS: endometriotic stromal cells.

MMP-2: Matrix metalloproteinase-2, MMP-9: Matrix metalloproteinase-9, αSMA: alpha smooth muscle actin, Col-I: Type I collagen.

CTGF: connective tissue growth factor, FN: fibronection.

However, one major concern in targeting the Wnt/β-catenin pathway is the potential for side effects on stem cell maintenance and tissue homeostasis [58]. Close attention should be paid to potential side effects of in vivo use of the Wnt/β-catenin pathway inhibitors in patients with endometriosis in further studies.
